# Supplementary material for: Elevated CO2 and warming change the nutrient status and use efficiency of Panicum maximum Jacq
Source: PLoS One. 2020 Mar 13;15(3):e0223937. doi: 10.1371/journal.pone.0223937 (PMC7069640; doi:10.1371/journal.pone.0223937)
Supplement: S1 File — (DOCX) [file pone.0223937.s001.docx]

CO_2_ (µmol mol^-1^)

Daily average values of CO_2_ concentration in control (C), elevated CO_2_ (eC), elevated temperature (eT) and the combination of elevated CO_2_ and elevated temperature (eC+eT) treatments. The elevated level of CO_2_ (set point 590 µmol mol^-1^) was maintained from sunrise to sunset in eC and eC+eT treatments. The black bars indicate the average of CO_2_ concentration during the night period. The yellow bars indicate the average of CO_2_ concentration during the light period. n = 4.
