# Supplementary material for: Elevated CO2 and warming change the nutrient status and use efficiency of Panicum maximum Jacq
Source: PLoS One. 2020 Mar 13;15(3):e0223937. doi: 10.1371/journal.pone.0223937 (PMC7069640; doi:10.1371/journal.pone.0223937)
Supplement: S2 File — (DOCX) [file pone.0223937.s002.docx]

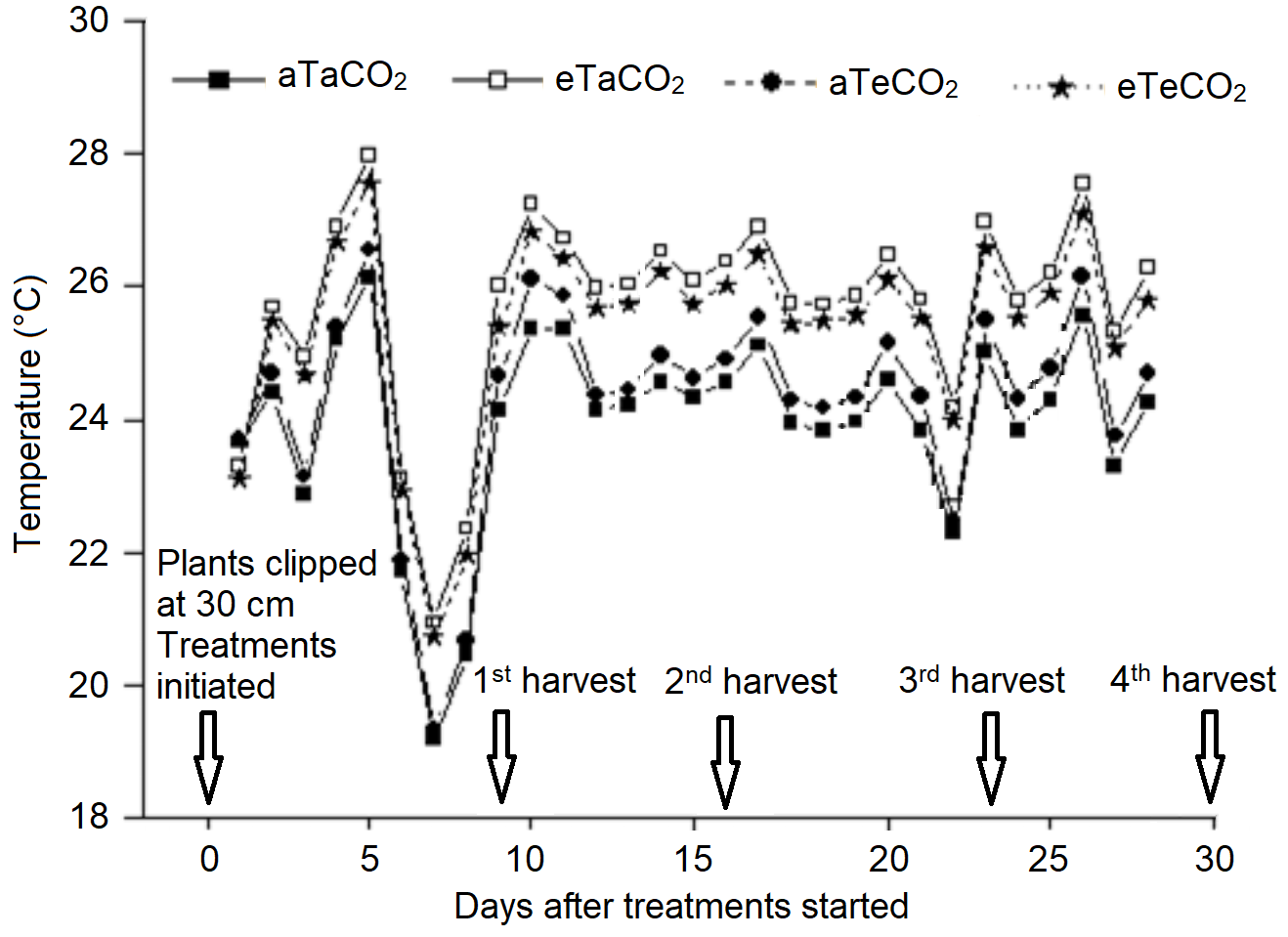


*P. maximum* canopy temperature during the treatments. Treatments: aTaCO_2_ (ambient temperature and ambient [CO_2_]), eTaCO_2_ (2°C above ambient temperature and ambient [CO_2_]), aTeCO_2_ (ambient temperature and 200 ppm above ambient [CO_2_]), and eTeCO_2_ (2°C above ambient temperature and 200 ppm above ambient [CO_2_]).
